# Cerium oxide nanoparticles-assisted aptasensor for chronic myeloid leukaemia detection

**DOI:** 10.5599/admet.2404

**Published:** 2024-08-18

**Authors:** Yuspian Nur, Muhammad Ihda HL Zein, Irkham Irkham, Shabarni Gaffar, Toto Subroto, Yeni Wahyuni Hartati

**Affiliations:** 1 Department of Chemistry, Faculty of Mathematics and Natural Sciences, Universitas Padjadjaran, Indonesia; 2 Laboratory of Research and Development of Farmaka Tropis, Faculty of Pharmacy, Universitas Mulawarman, Indonesia; 3 Research Center of Molecular Biotechnology and Bioinformatics, Universitas Padjadjaran, Indonesia

**Keywords:** Aptamer, K562 cell, screen-printed carbon electrode

## Abstract

**Background and purpose:**

Chronic myeloid leukaemia (CML) is one of the most lethal types of leukaemia and can rapidly progress if not treated properly. Therefore, having an effective diagnostic strategy is crucial. Various methods are available for diagnosis, including electrochemical biosensors with aptamer bioreceptors.

**Experimental approach:**

In this study, we immobilized the KK1D04 aptamer on a screen-printed carbon electrode (SPCE) supported by CeO_2_ nanoparticles (CeO_2_NPs) to detect K562 cells, a type of CML cell line. Several parameters were optimized to enhance the aptasensor response using the Box-Behnken experimental design.

**Key results:**

The developed aptasensor demonstrated good performance with a limit of detection (LOD) and limit of quantification (LOQ) of 16 cells/mL and 3,882 cells/mL, respectively, in the K562 cell concentration range of 10^2^ to 10^6^ cells/mL. The optimum experimental conditions were an aptamer concentration of 0.8 ppm, an aptamer incubation time of 36 minutes, and a K562 aptamer-cell incubation time of 13 minutes. The aptasensor also exhibits selectivity for K562 cells compared to Vero cells, THP1 cells, and Raji cells.

**Conclusion:**

The aptasensor in this study demonstrated the potential to detect K562 cells. These results could contribute to the advancement of point-of-care (POC) devices for the detection of CML.

## Introduction

Chronic myeloid leukaemia (CML) is a clonal myeloproliferative disorder that is caused by a genetic translocation of Abelson murine leukaemia (ABL) chromosome 9 and 22 breakpoint cluster region (BCR), which is known as the Philadelphia chromosome (Ph). The Ph chromosome generates the BCR-ABL1 gene fusion, which encodes a constitutively active tyrosine kinase so as leukemogenesis occurs. This disrupts downstream signalling pathways, leading to increased cell proliferation, lack of differentiation, and resistance to cell death. Some common symptoms of CML include anaemia, fatigue, weight loss, malaise and pain or fullness in the left upper quadrant of the abdomen [1-3]. Effective treatment is crucial to prevent rapid phase transfer in patients with CML. When patients enter the blastic phase, their survival rate is significantly reduced [4]. Abnormal cell proliferation in CML patients leads to the expression of several proteins that are not produced by normal cells. These proteins can potentially be used as diagnostic tools for detecting CML [5].

Since the introduction of imatinib in 2000, the annual mortality rate of patients with CML has significantly decreased from 10-20 % to 1-2 % 1. Other therapeutic drugs, such as nilotinib, dasatinib, bosutinib, and ponatinib, have also been used for CML therapy [6,7]. According to Rinaldi and Winston’s report, therapeutic drugs have shown promising results in curing CML patients and achieving treatment-free remission rates ranging from 28 % to 73 % in many countries [8]. The discovery of these drugs provides hope for patients to recover from CML disease. Therefore, it is crucial to develop a diagnostic strategy to ensure that patients receive appropriate therapy.

Electrochemical biosensors are a highly sensitive and portable diagnostic technique developed due to their fast analysis time and compact integration between bioreceptors and analytical devices [9]. Bioreceptor modification on the electrode can make it specifically detect the targeted analyte. Aptamers are one of the potential molecular bioreceptors used in the development of diagnostics [10-12]. Aptamers are promising bioreceptors due to their sensitivity, selectivity and low detection limit in biosensors [13-15]. Aptamers are synthetic single-stranded oligonucleotides (DNA or RNA) that can specifically bind to targets with high affinity [16]. The advantage of aptamer-based technology is the unique cell-based selection process. This process allows efficient selection of cell-specific aptamers without prior knowledge of the target [17]. Some interactions may occur when the aptamer interacts with the target, such as hydrogen bonding, electrostatic and hydrophobic interaction, Van der Waals interaction, structural compatibility, and aromatic ring stacking [18].

Utilization of cerium can enhance sensitivity and assist in immobilizing the aptamer on the electrode. Cerium in nanoparticle form (CeO_2_-NPs) possesses optical properties and biocompatibility, high mechanical strength, high thermal and chemical stability, oxygen storage capacity, and excellent electrocatalytic and biosensing properties [19-22]. The high biocompatibility of cerium oxide nanoparticles (CeO_2_-NPs) has facilitated their widespread application in the biomedical field. Several studies have demonstrated that CeO_2_-NPs can act as antioxidants and contrast agents for imaging [23,24]. The catalytic activity and therapeutic effects of CeO_2_-NPs are significantly influenced by their morphology [25]. The reversible conversion between Ce^3+^ and Ce^4+^ on the surface of the nanoparticles imparts exceptional redox properties. This allows CeO_2_-NPs to interact with reactive oxygen species (ROS), thereby reducing oxidative stress and enhancing self-regenerative capabilities. This property is one of the factors contributing to the increased biocompatibility of CeO_2_ [26]. Owing to this biocompatibility, CeO_2_-NPs hold potential for application with bioreceptors in biosensors, as they are considered non-toxic and capable of interacting with biological materials. Furthermore, the other study used CeO2-NPs in various biosensor applications [27-29]

There are various methods for immobilizing biomolecules, but one of the most effective and commonly used techniques is the streptavidin-biotin system [30]. This system relies on the strong non-covalent interaction between streptavidin and biotin, one of the strongest in nature. Streptavidin can bind to four biotins with a dissociation constant of 10^-15^ M^-1^. Biotin is also a stable molecule easily covalently bound to most biomolecules. As a result, a wide range of commercial biotinylated molecules can be used for biosensor applications. When a biotinylated molecule binds to streptavidin, the number of molecular conjugates on the probe increases, improving quality [31,32].

In this study, an aptasensor is made from screen-printed carbon electrode (SPCE), CeO_2_-NPs, and aptamer. The KK1D04 aptamer used in this study was previously used by Sefah *et al.* [33]. This aptamer specifically binds to the K562 cell (CML cell line). The aptasensor is expected to detect K562 cells with high sensitivity and selectivity, providing accurate analysis results, low detection limits, and rapid and simple analysis procedures compared to previous studies.

## Experimental

### Apparatus

The SPCE (Poten, China) was used as the electrode setup, consisting of a carbon electrode as the working and auxiliary electrode, with anAg/AgCl serving as the reference electrode for the electrochemical transducer. The electrochemistry measurements were conducted using a Sensit Smart potentiostat connected to a smartphone using PStouch v2.8 software (PalmSens BV, Netherlands). A scanning electron microscope (SEM) (FEI Inspect F50, USA) was used for electrode surface morphology analysis. Transmission electron microscopy (TEM) (Tecnai TEM120), UV-Vis spectrophotometer (Thermo Scientific, US), and particle size analyser (PSA) (BeckmanLS 13 320) were used for the characterization of CeO_2_.

### Chemicals

Cerium(III) nitrate hexahydrate 99 % trace metals basis, potassium ferricyanide (K_3_[Fe(CN)_6_]), and bovine serum albumin was purchased from Sigma Aldrich. Polyethylene glycol (PEG) from Clorogreen Gemilang, water for injection from IPHA Laboratories, streptavidin from Promega, biotinylated aptamer (biotin 5'- TEG- ATC CAG AGT GAC G CA GCA CAA AGT CTC TTC GGC GCG AAT CAG TTC ATC TTT CCC TGA TGG GGG TGG ACA CGG TGG CTT AGT-3') from Bioneer, potassium chloride (KCl), hydrogen peroxide 30 %, and phosphate-buffered saline (PBS) pH 7.4 from Merck. K562 (ATCC CCL-243), THP-1 (ATCC TIB-202), Raji (ATCC CCL-86), and vero cell lines were purchased from the American Type Culture Collection (ATCC, Manassas, VA). RPMI 1640 Medium, fetal bovine serum, and penicillin-streptomycin from Gibco. The K562 cells were cultured in RPMI 1640 media supplemented with 10 % fetal bovine serum (FBS) and 1 % penicillin-streptomycin and maintained at 37 °C under a humidified atmosphere containing 5 % CO_2_. The K562 cells were sub-cultured by replacing the fresh medium every 2 to 3 days under standard conditions as recommended by the supplier.

### Preparation of cerium oxide nanoparticle

Nanoparticle synthesis was adapted from Neal *et al.* [34]. The PEG polymer (2 g) was dissolved in 10 mL of distilled water and stirred until completely dissolved. Then, cerium nitrate powder was added to the PEG solution slowly while stirring vigorously. The resulting solution was stirred for 60 minutes, after which hydrogen peroxide (30 % solution) was dropwise and stirred again for 120 minutes. CeO_2_ was characterized using UV-VIS spectrophotometry and TEM.

### Modification of SPCE surface by CeO_2_-NPs and the characterization

The SPCE modification was carried out using the drop-casting method. The SPCE was first rinsed with demineralized water for pretreatment. Then, a solution of CeO_2_-NPs/PEG (40 μL) was dropped onto SPCE and incubated for 3 h at room temperature. Afterwards, the SPCE was rinsed again with demineralized water and dried at room temperature. The SPCE was characterized before and after modification using SEM (FEI Inspect F50, USA) and differential pulse voltammetry (DPV) with the redox system of [Fe(CN)_6_]^3-/4-.^

### Aptamer immobilization on the surface of CeO_2_-NPs modified SPCE

The immobilization of aptamers on SPCE was conducted using passive adsorption. Streptavidin (20 μL) was added to the surface of CeO_2_-NPs modified SPCE. The electrode was incubated for 50 minutes at 8°C. The incubated SPCE was then rinsed with PBS buffer solution pH 7.4. Next, the aptamer-biotin solution (20 μL) was dripped on the SPCE and incubated again at 8 °C. The SPCE was then rinsed with PBS buffer solution pH 7.4. Finally, the aptasensor was characterized using SEM and DPV with the redox system of [Fe(CN)_6_]^3-/4-^.

### Determination of aptasensor response to K562 cells

The site of SPCE that did not bind to streptavidin was blocked using 1 % BSA solution and incubated for 10 min at room temperature. The electrode was then washed with PBS buffer solution pH 7.4 and dried. After that, the K562 cell solution (20 μL) with varying concentrations was added to the electrode and incubated for 30 minutes at 37 °C. Each concentration was measured using differential pulse voltammetry with a redox system of 10 mM K_3_[Fe(CN)_6_] solution in 0.1 M KCl over a potential range of -0.3 to +0.5 V with a scan rate of 0.008 V/s.

### Optimization of parameters affecting the experiment with Box-Behnken design

The parameters selected for optimization were aptamer concentration (*X*_1_), aptamer incubation time (*X*_2_), and KK1D04 aptamer incubation time with K562 cells (*X*_3_). Each parameter was designed with 3 different levels, which are the lowest (-1), middle (0), and highest (+1) levels, as provided in Table 1. The measurement responses from the suggested experiments were then processed, and the optimum value of each parameter was determined using the Box-Behnken experimental design with the Minitab 21 program.

## Results and discussion

### Characterization of CeO_2_ nanoparticles

Over the years, there has been a growing interest in the unique catalytic properties of cerium oxide nanoparticles (CeO_2_-NPs). These nanoparticles possess stable mechanical properties, biocompatibility, and distinctive catalytic properties, making them suitable for various applications in biotechnology, agriculture, and biomedicine [25]. Cerium exhibits an outstanding cycling character in two ionic states, Ce^3+^ and Ce^4+^, due to the presence of electrons in the ground state in the 4f orbital (Xe 4f^1^ 5d^1^ 6s^1^), which demonstrates the redox properties of cerium [35]. The sensor was developed using the catalytic activity and oxygen exchange capacity of CeO_2_-NPs. The catalytic element of CeO_2_-NPs can enhance the signal, and they can also mimic the enzyme properties to displace biological enzymes as an alternative to more stable probes, as well as a transducer due to physicochemical properties that can bind analytes, measurable by optical or electrochemical devices [36].

**Figure 1.**
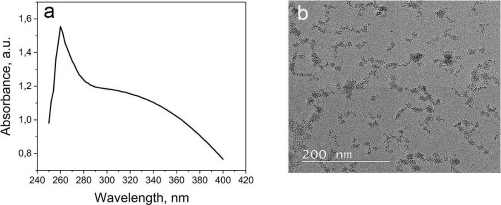
(a) UV Vis spectra of CeO_2_-NPs. (b) TEM image of CeO_2_-NPs.

The CeO_2_-NPs were synthesized using PEG as a template and CeO_2_ as a size stabilizer, along with H_2_O_2_ to oxidize Ce^3+^ to Ce^4+^. The UV-Vis spectrophotometer characterization of CeO_2_-NPs revealed the highest absorption at a wavelength of 260 nm (Figure 1A). The absorption band of CeO_2_-NPs falls in the range of 250 to 400 nm. The strong absorption band at lower 400 nm is due to the charge transfer transition from O^2-^ (2p) to Ce^4+^ (4f) orbitals on CeO_2_. It is important to note that the absorbance can be influenced by various factors, such as particle size, band gap, oxygen deficiency, grid strain, and surface roughness [37-39].

The morphology analysis of the synthesized CeO_2_-NPs was analysed using TEM (Figure 1B). Although CeO_2_ is evenly distributed, some aggregation is still observed at certain points, possibly due to the suboptimal ratio of cerium nitrate and PEG. The particle size of CeO_2_-NPs falls within the nanometer range, and the size of CeO_2_ particles was further confirmed using PSA, revealing a particle size range of 67-165 nm for CeO_2_-NPs.

The purpose of using PEG is to increase the solubility of CeO_2_ (PEG-lytion) and to prevent the synthesized CeO_2_-NPs from aggregating. PEG is adsorbed on the surface of CeO_2_ through physical interaction, which helps to maintain the interaction between CeO_2_. Moreover, PEG is stable against strong oxidizers such as hydrogen peroxide [34,40].

### Aptasensor characterization

**Figure 2.**
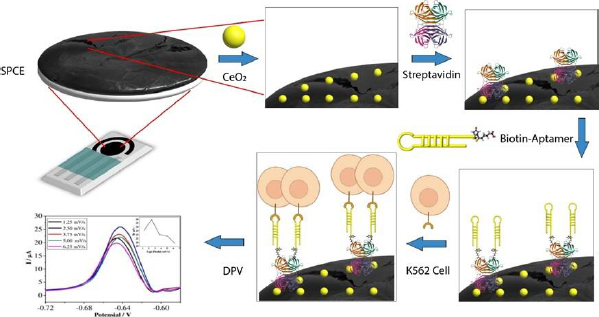
Schematic illustration of CeO_2_-NPs modified aptasensor for K562 cell detection.

Carbon-based SPCE is used for the quantitative detection of analytes to replace some instruments. SPCE-based electrochemical techniques have several advantages, such as rapid, miniaturized, and easy to use. SPCE is easy to mass-produce and modify, making it a preferred electrode type compared to others [41]. The use of cerium-modified SPCE can increase the current during measurement. The conductivity of cerium can enhance the electroactive properties that play a role in electron transfer, as well as binding to certain proteins or bioreceptors [14,39,42]. Figure 2 illustrates the scheme of SPCE modification using CeO_2_-NPs and biological elements to detect K562 cells. The electrochemical biosensor for detecting K562 cells works by measuring a redox system of K_3_[Fe(CN)_6_] 10 mM in 0.1 M KCl. K_3_[Fe(CN)_6_] is used because it has a sensitive electrochemical response on the carbon surface [43]. The current generated is proportional to the number of cells detected.

SPCE was first modified with CeO2-NPs to prepare the biosensor using the drop-casting method. Then, the KK1D04 aptamer was immobilized on the modified SPCE. Streptavidin was added to the modified SPCE to bind the biotinylated KK1D04 aptamer. The interaction between streptavidin and biotin is one of the strongest interactions. The interaction between the K562 cells and KK1D04 aptamer has a binding affinity of 10^-15^ M^-1^ [44].

To ensure accurate measurement of the analyte, the electrode was treated with BSA prior to measuring the number of K562 cells. This treatment helps to block the current in measurement and prevent any bias in the measurement of the analyte [45]. After each modification step, the excess or unimmobilized molecules added were removed by rinsing the electrode. The final step involved adding SPCE modified with KK1D04 aptamer to a number of K562 cells and incubating them at 37 °C. The K562 cells will interact specifically with the KK1D04 aptamer, producing a low electrochemical signal from K_3_[Fe(CN)_6_]. If no K562 cells interact with the KK1D04 aptamer, the electrochemical signal of K_3_[Fe(CN)_6_] will be high.

**Figure 3.**
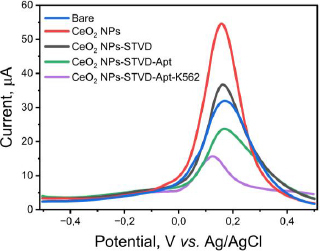
DPV curve from each level of SPCE modification up to K562 cell assay.

The pre- and post-modified SPCEs were characterized using differential pulse voltammetry to determine the success of the modification. The measurements were conducted using a redox system K_3_[Fe(CN)_6_] 10 mM in 0.1 M KCl. The results of the DPV method showed an increase in current post-modification (Figure 3). The current generated by the CeO_2_-NPs modified SPCE was 54.368 μA (red line), whereas the bare SPCE produced only 31.410 μA (blue line) before modification. The current increase was recorded to be nearly two-fold. The drop casting technique used to modify the SPCE/CeO_2_-NPs can improve the electron transfer process, making the electrode more sensitive [26,46]. Furthermore, the use of CeO_2_-NPs can enhance the biocompatibility of the electrode, making the electrode modification process simpler. One example of this is the use of the biotin-streptavidin system. The addition of streptavidin to the electrode caused a decrease in current to 36.015 μA (black line) in SPCE/CeO_2_-NPs/STVD. Streptavidin attaches to the electrode due to the electrostatic interaction between CeO_2_-NPs, which has a high affinity with the -NH_2_ group on streptavidin. CeO_2_-NPs are small, resulting in a large surface area capable of binding molecules such as amino acids, proteins, and other biological molecules. Additionally, CeO_2_-NPs can interact with the carboxyl functional groups of antibodies without the need for additional binding agents [14]. Furthermore, the addition of biotinylated KK1D04 aptamer also caused a decrease in current to 21.534 μA (green line) due to the addition of non-electroactive biomolecules. Similarly, the addition of K562 cells caused a decrease in current to 10.745 μA (purple line). All current decreases occurred due to the addition of large and non-electroactive molecules or biomolecules, which inhibited the transfer of ferricyanide species electrons on the electrode surface [14]. This decrease in current indicates that each stage of modification and measurement of K562 cells was successfully carried out.

**Figure 4.**
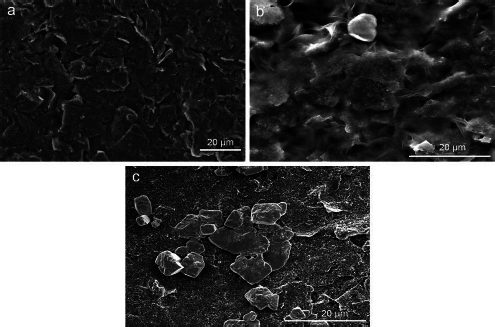
SEM image of SPCE modification: (a) SPCE bare, (b) SPCE/CeO_2_-NPs, (c) SPCE/CeO_2_-NPs/streptavidin/aptamer.

The SEM images of the working electrode surface of the SPCE before and after modification were analysed to determine the morphology, as shown in Figure 4. The bare SPCE surface in Figure 4A appears smooth and homogeneous, with no particles except for carbon. Figure 4B shows the SEM image of the CeO_2_-NPs modified SPCE surface, which appears rougher with a layer of CeO_2_-NPs synthesized with PEG. Although the layer is not evenly spread and there are some aggregated parts, it can still enhance the conductivity of the carbon electrode and provide numerous active sites for the immobilization of streptavidin and KK1D04 aptamer. Figure 4C shows the successful immobilization of aptamer KK1D04 on the working electrode surface of the SPCE, which is distributed on the surface of SPCE/CeO_2_-NPs and will be used to detect K562 cells.

### Determination of optimum conditions with Box-Behnken experimental design

The optimum condition of the aptasensor to detect K562 was determined by the Box-Behnken design on Minitab 21. Three parameters that affected the performances of aptasensor were aptamer concentration (*X*_1_), aptamer incubation time (*X*_2_), and K562 aptamer-cell incubation time (*X*_3_). Incubation time is one of the important parameters in aptasensor manufacturing. The optimum incubation time determines whether or not the biomolecules and target analytes bind perfectly. However, in the application, we hope the aptasensor can detect the target analyte as rapidly as possible and produce accurate data. The effect of the response current of the three parameters was tested with DPV using a redox system of K_3_[Fe(CN)_6_] 10 mM in 0.1 M KCl at a potential range of -0.3 to 0.5 V with a scanning rate of 0.008 V/s. The correlation of the current response with each parameter is designed at three levels, *i.e*., the lowest level (-1), intermediate level (0), and the highest level (+1) shown in Table 1. The expected optimum response is the minimum current from the design results of the three parameters.

Based on the measurement results and data processing in the Minitab 21 program, the regression Equation (1) is obtained:


(1)

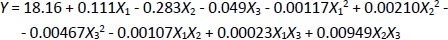



Equation (1) shows that parameters with positive values increase the current response, while parameters with negative values decrease the experimental current response. Table 2 shows the ANOVA results based on the resulting response data, with the p-value explaining the variable data. If the p-value is less than 0.05 (*p*-value < 0.05), a variable has a linear effect. The p-value is also used to determine the significance of each variable and the interaction effect of the combination of two variables [47]. Variables with a p-value above 0.05 (*p*-value > 0.05) are considered to have no significant effect.

According to Table 2, the parameters of aptamer concentration (*X*_1_), aptamer incubation time (*X*_2_), and K562 cell aptamer incubation time *(X*_3_) do not significantly affect the experimental results as their p-value is above 0.05. The correlation coefficient (*R*^2^ = 0.6799) suggests a medium correlation between the parameters. The linear model obtained is appropriate or acceptable with a p-value for lack of fit (*p* > 0.05) of 0.153 [39]. The steepest descent method was used to find the minimum value of a function, which in this case is the optimum current response generated by the K562 cell interacting with the KK1D04 aptamer on the electrode. The results of the steepest descent indicate that the lowest response value was obtained at an aptamer concentration of 0.8 ppm, aptamer incubation time of 36 minutes, and K562 aptamer-cell incubation time of 13 minutes, resulting in a current response of 13.484 μA.

According to Table 2, the parameters of aptamer concentration (*X*_1_), aptamer incubation time (*X*_2_), and K562 cell aptamer incubation time *(X*_3_) do not significantly affect the experimental results as their p-value is above 0.05. The correlation coefficient (*R*^2^ = 0.6799) suggests a medium correlation between the parameters. The linear model obtained is appropriate or acceptable with a p-value for lack of fit (*p* > 0.05) of 0.153 [39]. The steepest descent method was used to find the minimum value of a function, which in this case is the optimum current response generated by the K562 cell interacting with the KK1D04 aptamer on the electrode. The results of the steepest descent indicate that the lowest response value was obtained at an aptamer concentration of 0.8 ppm, aptamer incubation time of 36 minutes, and K562 aptamer-cell incubation time of 13 minutes, resulting in a current response of 13.484 μA.

### Determination of validation parameters

**Figure 5.**
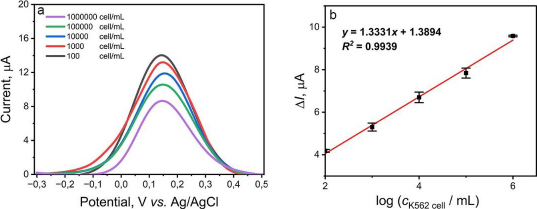
(a) DPV curve for a range of target K562 cells from 10^2^ to 10^6^ cells/mL; (b) Linear relationship between the change of current (Δ*I*) and the logarithm of the target K562 cells concentration. Error bars represent standard deviations obtained in three parallel experiments.

The validation parameters determined in this study were the limit of detection (LOD) and the limit of quantification (LOQ) of the aptasensor to detect K562 cells and provide the calibration curve. The variation of K562 cell concentration is measured in range from 10^2^ to 10^6^ cells/mL. A gradual decrease in current was observed as the concentration of K562 cells increased, as shown in Figure 5A. The K562 cells are not electroactive, so the more cells that bind to the KK1D04 aptamer on the aptasensor, the more inhibited electron transfer occurs. The value of Δ*I* versus the logarithm of K562 cell concentration showed a good correlation coefficient (*R*^2^) value of 0.9939, with the regression equation obtained as *y* = 1.3331*x* + 1.3894 (Figure 5B). The calculated LOD and LOQ values were 16 and 3,882 cells/mL, respectively. The detection limit of the aptasensor in this study is quite low compared to several studies for K562 cell detection, which can be seen in Table 3. The development of a biosensor requires precision and accuracy to be viable as a detection method. In this study, precision and accuracy were determined by measuring a single concentration of K562 cells with six repetitions. Based on the calculations, this aptasensor demonstrated precision and accuracy values of 98.30 and 98.45 %, respectively. The SPCE modified with CeO_2_-NPs and KK1D04 aptamer has the potential to be used and developed for K562 cell detection. The advantages of the aptasensor constructed in this work, compared to other sensors used (Table 3), include its relatively simple modification process, a low detection limit compared to various electrode modifications, and a broad linearity range from 10^2^ to 10^6^ cells/mL. Additionally, the detection time of this aptasensor is faster, approximately 13 minutes.

**Figure 6.**
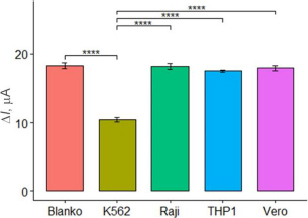
Comparison of DPV peak currents using different cells with a concentration of 10^5^ cells/mL. Error bars represent standard deviations obtained in three parallel experiments. ****(*p* < 0.05) indicates a significant difference in the current response between samples based on Anova and Tukey tests.

In this study, the selectivity of the aptasensor was tested against Vero cells, Raji cells, and THP1 cells at a concentration of 10^5^ cells/mL, with PBS as the blank. The selectivity test aimed to assess the aptasensor ability to specifically respond to and detect K562 cells, differentiating them from normal cells and other cell types. The tests were conducted in three parallel experiments. The current response of the aptasensor to each cell type was observed, and the selectivity was considered satisfactory if the aptasensor exhibited a significant response only to K562 cells and not to the other cells. As expected, Figure 6 shows high currents on the Vero, Raji, and THP1 cells, similar to the blank. The results of the ANOVA and Tukey tests indicated a significant difference with a p-value below 0.05 among the K562 cells and the other. These results confirm that the aptasensor with aptamer KK1D04 as a receptor is selective to K562 cells.

An aptasensor is a type of biosensor known for its good reproducibility and stability. This has been demonstrated in several reported studies [48-50]. The aptasensor maintains storage stability for 30 days at 4 and 25 °C, retaining 82-95.3 % of its measurement activity. With these results, the aptasensor shows promise as a detection tool for chronic myeloid leukaemia (CML).

## Conclusions

Aptasensor combines the advantages of electrochemical sensors and aptamers used to detect leukaemia, especially chronic myeloid leukaemia (CML). The development of this aptasensor, the use of CeO_2_-NPs precipitated on the surface of the working electrode from SPCE, can increase conductivity and play a role in binding to certain proteins or bioreceptors. The synthesis of CeO_2_-NPs with PEG and H_2_O_2_ produces particle sizes in the range of 67-165 nm. Meanwhile, the performance of aptasensor showed a linear range from 10^2^ to 10^6^ cells/mL with high linearity (*R*^2^ = 0.9939) and a quite low LOD of 16 cells/mL. The detection of the K562 cell was performed quite fast at 13 minutes, but it is not the optimum time for this aptasensor. This aptasensor also exhibited good selectivity for K562 cell detection. As a complement to the RT-PCR method, this aptasensor has the potential for early detection of CML disease diagnosis. In summary, the proposed aptasensor enables the fabrication and development of electrochemical biosensors that should be further developed and applied to clinical samples.

## Figures and Tables

**Table 1.** Optimization of parameters affecting experimental conditions using Box-Behnken.

Parameters	Level		
	-1	0	+1
Aptamer concentration, μg/mL (*X*_1_)	0.2	0.5	0.8
Aptamer incubation time, min (*X*_2_)	10	30	50
Aptamer - K562 Cell incubation time, min (*X*_3_)	4	17	30

**Table 2.** Analysis of variance.

Source	DF	Adj SS	Adj MS	F-Value	P-Value
Model	9	61.9452	6.8828	1.18	0.451
*X*_1_	1	8.4851	8.4851	1.45	0.282
*X*_2_	1	7.4209	7.4209	1.27	0.311
*X*_3_	1	10.5111	10.5111	1.80	0.237
*X*_1_^2^	1	4.0876	4.0876	0.70	0.441
*X*_2_^2^	1	2.6167	2.6167	0.45	0.533
*X*_3_^2^	1	2.3039	2.3039	0.40	0.557
*X*_1_ *X*_2_	1	1.6358	1.6358	0.28	0.619
*X*_1_ *X*_3_	1	0.0312	0.0312	0.01	0.945
*X*_2_ *X*_3_	1	24.3690	24.3690	4.18	0.096
Error	5	29.1615	5.8323		
Lack-of-Fit	3	26.0957	8.6986	5.67	0.153
Pure Error	2	3.0658	1.5329		
Total	14	91.1067			

**Table 3.** Comparison with other biosensors for K562 cells detection.

Methods	Aptamer	Sensing method	Linear range of target analytes, cell mL^-1^	LOD, cell mL^-1^	Ref.
SPCE/NPG/Aptamer with ZnO@CQDs label	KK1B10	Electrochem-iluminescence	10^2^ to 2×10^7^	46	[51]
G-quadrup006Cex DNAzyme for adenosines, H_2_O_2_, Fe_3_O_4_ nanoparticle and MB	KK1B10	Electrochemical	14 to 14×10^6^	14	[52]
GCE/PtNPs with gold cage/Ru(bpy)_3_^2+^-labeled concanavalin A	KK1B10	Electrochem-iluminescence	500 to 5×10^6^	500	[53]
Gold electrode/Aptamers and bio-ConA label	KK1B10	Electrochemical	10^2^ to 10^7^	79	[54]
SPCE/CeO_2_-NPs/Aptamers	KK1D04	Electrochemical	10^2^ to 10^6^	16	This work
